# Developing a model of quality physical education in the Chinese context: a grounded theory investigation of secondary school physical education teachers’ perceptions

**DOI:** 10.3389/fpubh.2025.1569222

**Published:** 2025-05-01

**Authors:** Ling Qin, Walter King Yan Ho, Bing Xu, Selina Khoo

**Affiliations:** ^1^Faculty of Physical Education and Health Science, Chongqing Normal University, Chongqing, China; ^2^Faculty of Sports and Exercise Science, Universiti Malaya, Kuala Lumpur, Malaysia; ^3^Faculty of Education, Tokyo Gakugei University, Tokyo, Japan

**Keywords:** quality physical education, secondary school, teacher, grounded theory, model development, China

## Abstract

**Background:**

Quality Physical Education (QPE) programs have received increasing attention as a means to address the significant decline in adolescents’ physical health. Since the introduction of the general concept of QPE by the United Nations Educational, Scientific and Cultural Organization in 2015, there has been limited research on QPE within non-Western areas, particularly in China. This research gap hinders the development of QPE strategies and practices tailored to China’s specific context, which is crucial for improving adolescents’ health. Therefore, this study aimed to develop a model of QPE in China.

**Method:**

This qualitative study adopted a grounded theory approach to examine Chinese physical education (PE) teachers’ perspectives on quality PE. Twenty-two PE teachers from diverse regions were purposively sampled and in-depth interviews were conducted online. Interview transcripts were recorded, transcribed verbatim, and analysed following open, axial, and selective coding.

**Results:**

The QPE model consisted of five levels, 12 categories, 51 concepts, and 216 statement labels. The five levels are: (1) student level (comprising students’ development, and students’ engagement and experiences in PE), (2) family level (comprising parents’ engagement and attitude toward PE, and home-based sports resources), (3) school level (comprising PE teacher, sports facilities and equipment in the school, PE curriculum, school-based extracurricular PA programs, co-operation family-school-community in PE, and school leadership and school community support for PE), (4) community level (comprising community-based sports resources), and (5) government level (comprising government support for PE).

**Conclusion:**

This study broadens global knowledge of QPE, particularly in non-Western countries. It provides practical implications for policymakers and educators in secondary education to design and improve QPE programs. By fostering a sustainable educational framework. These contributions are essential for developing effective strategies that promote PA engagement and well-being among adolescents.

## 1 Introduction

The World Health Organization (WHO) classifies individuals aged 10 to 19 years as adolescents, a unique group that begins to form independent lifestyle habits which may persist into adulthood (1). Unfortunately, the global prevalence of overweight and obesity in this age group is rising. Zhang et al.’s (2) study indicated that 158 million children and adolescents aged 5 to 19 years were obese in 2020. This number is projected to increase to 206 million by 2025 and to 254 million by 2030 (2). Excess weight during adolescence is associated with negative health outcomes later in life, including cardiovascular diseases and type 2 diabetes (3). Therefore, early intervention is crucial to prevent future chronic diseases and related health complications. Regular physical activity (PA) plays a critical role in fostering the physical, psychological, emotional, and cognitive development of children and adolescents, contributing to their overall health and well-being (4). It is widely recognized that engaging in sufficient PA is essential for maintaining physical fitness, preventing chronic diseases (5), and supporting mental health by reducing symptoms of anxiety, depression, and stress (6). Furthermore, regular PA has been linked to improved academic performance (7), as it enhances cognitive functions such as concentration, memory, and problem-solving skills (8). Despite these well-documented benefits, global data indicate that only 27 to 33% of children and adolescents meet the recommended 60 min of moderate to vigorous PA each day (9). Research demonstrates that PA engagement begins to decline during childhood and continues to decrease through adolescence (10). A national survey in China assessed PA levels among 133,006 school-aged children and adolescents aged 9 to 17 years, using the Global Matrix 4.0 Indicators framework (11). The study revealed significantly low PA levels among Chinese youth. Only 14% met the WHO’s guidelines in 2022 (12). Therefore, supporting adolescents in enhancing their PA is essential for reducing the global increase in overweight and obesity rates among this population.

Quality Physical Education (QPE) programs have gained increasing attention as a means of addressing the significant deterioration of physical health in adolescents (13–15). QPE was first introduced on the National Association for Sport and Physical Education website (16). It emphasized the importance of addressing curriculum development, innovative teaching approaches, and essential support factors, including sufficient instructional time, facility enhancement, human resource allocation, and policy advocacy. Masurier and Corbin (17) outlined 10 reasons for QPE in schools, such as its ability to provide unique opportunities for PA, promote lifelong physical fitness and wellness, and educate the total person. Similarly, Ball et al. (18) contended that a well-implemented QPE program can significantly benefit students by increasing MVPA levels. Moreover, QPE programs are designed to foster positive attitudes toward PA, promoting the enjoyment of movement and the development of physical skills that students can use throughout their lives (19). Notably, to provide practice guidelines for the practice of QPE, the United Nations Educational, Scientific and Cultural Organization (UNESCO) has established a global definition of QPE as (p 9.) ‘the planned, progressive, inclusive learning experience that forms part of the curriculum in early years, primary, and secondary education’ (14). UNESCO emphasizes inclusive approaches, curriculum flexibility, and multistakeholder involvement, such as schools, communities, and governments. These efforts illustrate a trend toward understanding QPE in broader and more holistic terms, aligning with wider educational objectives (20). This conceptualization has been influential in shaping physical education (PE) programs in Western education systems. National policies in Canada, Australia, and the United Kingdom (UK) demonstrate how UNESCO’s guidelines have been adapted to meet local educational needs. Canada’s Physical and Health Education curriculum outlines a K-12 structure emphasizing diverse physical activities, holistic assessments, and active student participation, aiming to develop lifelong PA skills (21). In Australia, QPE is defined by four core components: the minimum allocated time for physical activities, alignment with curriculum standards, employment of specialized PE teachers, and creation of safe and inclusive environments for students (22, 23). Similarly, in the UK, QPE is characterized by intelligent movement, inclusivity, knowledge promotion, effective assessments, and teacher expertise, all of which aim to foster active and healthy lifestyles in children and adolescents (24).

Despite advancements in implementing QPE programs in Western countries, the interpretation of the term ‘quality’ remains highly variable. In general, ‘quality’ is defined in numerous ways, such as excellence (25), value (26), fitness for use (27), conformance to requirements (28), and meeting or exceeding expectations (29). Although these definitions often overlap, there is no consensus on the definitive interpretation of the term. In the context of QPE, this lack of definitional clarity poses challenges across disciplines, including PE, education, psychology, and public health sciences (30). This ambiguity complicates the practical implementation of QPE, particularly in non-Western countries, where local contexts and educational frameworks differ significantly from Western models. For example, Uhlenbrock and Meier (31) argued that UNESCO’s QPE guidelines, although globally influential, have not been adequately adapted to address specific educational systems and community experiences in non-Western countries. This highlights the need for QPE to be implemented with greater sensitivity in local contexts to ensure its success as a collaborative and culturally attuned initiative.

China offers a distinctive context for examining QPE because of its unique educational system. This system operates under a centralized policy framework. In addition, China’s emphasis on reforming PE encourages students to establish healthy lifestyles and values. PE in China has been a mandatory component of the national curriculum since 1949, with the Soviet Union’s performance-oriented model of sports skills deeply influencing the country’s early PE practices (32). In the early years, PE was primarily designed to serve national defense purposes (33). However, introducing the ‘quality education’ concept in 1998 marked a shift away from this old model. In the Ministry of Education’s *Action Plan for Invigorating Education in the 21st Century*, quality education became a key objective, emphasizing the importance of early childhood education, education for children with disabilities, and PE (34). This change led to significant reforms in China’s PE curriculum post-2000, including the Ministry of Education’s release of the *Physical Education Curriculum Standards for Compulsory Education (Grades 1–6)* and *High School Education (Grades 7–9)*, which prioritized a ‘health-first’ approach (33). China’s approach to improving PE quality has continued to evolve in recent years. In 2020, a directive was issued to promote a holistic PE framework involving the collaboration of families, schools, governments, and society to support students’ physical and mental well-being (35). This initiative was followed by a 2021 policy to reduce academic pressure on students and encourage more active participation in PE (36). The 2022 national PE curriculum standards further highlighted the government’s commitment to the ‘health-first’ principle, promoting daily exercise, inclusivity, and the development of healthy lifestyle habits (37). The standards also emphasize inclusivity, ensuring that all students benefit equally from PE programs, regardless of regional or individual differences. By 2023, the government shifted toward refining a collaborative educational mechanism led by schools and supported by families and communities (38). This approach underscores the importance of daily outdoor activities and provision of professional guidance and accessible sports facilities, reflecting a continued dedication to advancing the quality of PE.

While the Chinese government has introduced various policies to improve PA and promote the inclusivity and quality of PE, such as those outlined in UNESCO’s guidelines (14), the specific objectives and implementation strategies are often not explicitly addressed or clearly defined in China. This lack of clarity creates challenges in translating these broad macro-level goals into practical and actionable school implementation strategies. Moreover, despite the studies in the field of QPE being conducted in different countries (20, 39, 40), there is a noticeable gap in research on QPE within the Chinese context. Furthermore, China has the world’s largest secondary education system, with 80.4732 million secondary school students and 67,700 schools (41). Therefore, understanding secondary school PE teachers’ perspectives on QPE is essential. These teachers are central to delivering QPE and play a crucial role in shaping students’ PE experiences through pedagogical strategies and involvement in learning (42, 43). As key stakeholders in QPE implementation, their insights are invaluable for informing policy development and ensuring that QPE programs are effective and meaningful. Finally, as physical health among the youth has declined dramatically in recent years (44), this study offers significant guidance on enhancing the PE quality, aiming to improve adolescents’ physical health.

This study addresses key research gaps by examining the perspectives of secondary school PE teachers in China on QPE. Its objective is to develop a comprehensive, context-specific QPE model in China grounded in PE teacher insights. A research question in this study is: What are the levels and categories of QPE in China? Given the complexity of QPE as a social phenomenon, this study adopts a grounded theory approach that is well-suited for exploring diverse, context-specific experiences (45). Grounded theory has been widely used in related fields, such as PA (46), PE curriculum (47), and teacher professional development (48), and offers a robust framework for capturing the nuanced views of Chinese PE teachers. This study explores these perspectives to provide the model for developing QPE in China. It contributes to the creation of a sustainable educational framework aimed at improving adolescents’ physical health.

## 2 Materials and methods

**Figure 1:**
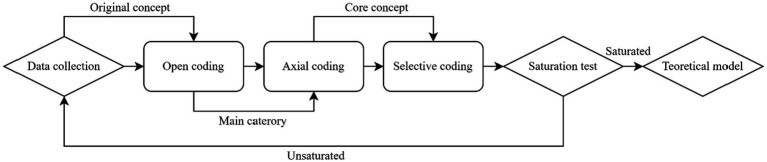
Process of grounded theory (83).

Grounded theory is a qualitative research approach that emphasizes theory development through a bottom-up process, avoiding preconceived notions and ensuring greater objectivity in the resulting theoretical model (49). This method is distinguished by its practice-oriented focus, reliance on empirical evidence, and foundation in real-world experience. As a qualitative research method, grounded theory enables researchers to deeply explore the experiences, beliefs, and emotions of research subjects, facilitating a more nuanced understanding and interpretation (50). The bottom-up approach in grounded theory ensures that the resulting theories are thorough and authentic (49). Consequently, models generated using grounded theory are often more complex, providing better practical guidance and suitability for constructing theoretical frameworks (45). The reliability and accuracy of the original data play a critical role in determining the quality of the outcomes in grounded theory research (51). These characteristics are particularly valuable in the current context because there is a lack of research on QPE in China. Grounded theory can establish a comprehensive awareness of the specific categories of QPE, enabling rapid immersion in QPE practice. The grounded theory process is illustrated in Figure 1.

This study was conducted from November 2022 to March 2023. Ethical approval was obtained from the Universiti Malaya Research Ethics Committee (UM.TNC2/UMREC_2042), and in China, approval was granted by Chongqing Normal University. Written informed consent was obtained from the school principals and PE teachers.

### 2.1 Participants

Participants were PE teachers from junior and senior secondary schools in China’s eastern, western, central, northern, and southern regions (see Table 1). The interview process was recorded with the participants’ permission. Inclusion criteria for PE teachers included (a) In-service PE teachers in secondary schools, (b) teachers representing all age groups and varying lengths of teaching experience, and (c) willingness to participate in this study with a sign-on consent form. A total of 22 PE teachers (16 males and 6 females) were included. Their ages ranged from 25 to 57 years (*M* = 38.23, SD = 8.40), and their teaching experience ranged from 2 to 36 years (*M* = 16.23, SD = 9.03). We employed a purposive sampling method to recruit PE teachers for the in-depth interviews. Thomson (53) recommended that 20 interviews were sufficient for grounded theory.

**Table 1:** Summary of PE teachers characteristics.

Surname	Gender	Age	Years of work	Type of school	Region
Wang	Male	43	20	Senior Secondary School	Southern China
Sheng	Male	28	6	Senior Secondary School	Northern China
Li	Male	34	12	Senior Secondary School	West China
Su	Male	37	14	Senior Secondary School	West China
Pang	Male	25	2	Senior Secondary School	West China
Huang	Male	34	12	Senior Secondary School	West China
P. Chen	Male	36	13	Senior Secondary School	Northern China
L. Ma	Male	35	14	Senior Secondary School	Southern China
He	Male	51	30	Senior Secondary School	Southern China
Y. Ma	Male	48	29	Senior Secondary School	Northern China
Tan	Male	37	14	Junior Secondary School	West China
Yao	Male	25	3	Junior Secondary School	West China
Tian	Male	36	11	Junior Secondary School	Eastern China
Feng	Male	46	22	Junior Secondary School	Southern China
Liang	Male	30	7	Junior Secondary School	West China
Dai	Male	32	12	Junior Secondary School	Northern China
Liu	Female	52	30	Senior Secondary School	West China
Gao	Female	42	22	Senior Secondary School	Central China
Long	Female	41	19	Junior Secondary School	Eastern China
Meng	Female	39	17	Junior Secondary School	Central China
Yang	Female	57	36	Junior Secondary School	Eastern China
XY. Chen	Female	33	12	Junior Secondary School	West China

### 2.2 Data collection

The interview questions were developed based on a thorough review of relevant literature (14, 16, 21, 24, 52). Before conducting the interviews, insights into the interview questions were gathered from three experts (a senior-level PE teacher and two experts in PE and education). Their expertise ensured that the questions closely aligned with the research objectives. Ultimately, this study designed four main open-ended questions (see Table 2) focusing on three key areas: (a) interviewees’ experiences in PE, (b) the advantages and challenges in the quality of PE, and (c) the dimensions of QPE. Owing to geographical constraints, interviewee preferences, and the COVID-19 lockdown policy in China, all interviews were conducted online.

**Table 2:** Interview questions.

No.	Questions
1	Could you describe your personal experiences and observations while teaching PE classes?
2	In your opinion, what aspects contribute to improving the quality of PE? What areas do you think need further improvement?
3	What do you consider to be the primary challenges in implementing PE? How do these challenges impact the effectiveness of PE teaching?
4	Based on your experience, what key dimensions or elements should QPE include?

### 2.3 Data analysis

Following the interviews, the audio recordings were transcribed into text using Microsoft Word. The transcriptions were carefully reviewed to ensure accuracy and focus on the content relevant to the objectives of the study. A total of 22 transcripts were generated from the in-depth interviews, comprising 64,102 words. These data were analysed using the three-level coding approach of grounded theory with NVivo 12 software, involving open, axial, and selective coding.

#### 2.3.1 Open coding

Open coding represents the initial phase of analysing interview data, in which raw empirical information is conceptualized, refined, and categorized (49), particularly regarding participants’ perspectives on QPE. This process involves identifying and labelling relevant statements while removing irrelevant content to minimize bias from subjective interpretations or prior research. Through this method, statements are systematically distilled into categories and labels, aiding in identifying underlying concepts.

Step 1: Labelling. This involves transforming interview data into labelled statements. A total of 216 labelled statements were generated (see Table 3). The labels indicate that the PE teachers focused on several aspects of the components of QPE in China, such as government policy in QPE, parents’ roles in QPE, and the quality of the PE curriculum.

**Table 3:** Labelled 216 statements from PE teacher interviews.

Tagged labels	Tagged labels
PE teachers should accompany and actively partake alongside students in physical activities and athletic competitions during PE.	Schools should offer PE teaching content with different sports options, and students should be able to choose classes based on their interests.
QPE should cultivate students’ awareness of the need to participate actively in PA.	Parents should support PE, recognizing that physical exercise benefits their children’s health and well-being.
After class, if students ask the PE teacher about areas they need to improve in sports, teachers should provide detailed coaching.	PE is challenged by the universalization and homogenization of its content.
School recess physical activities should be a fun and active experience for students.	High-quality PE teachers are essential for implementing high-quality PE.
Our school offers extracurricular sports training services, allowing students to select programs based on their interests and preferences.	QPE could enhance relationships among students.
PE teachers should respect students’ ideas.	PE teachers could improve the quality of teaching through PE focus group meetings.

For the specific data, see Supplementary Table S1.

Step 2: Conceptualization. By comparing, refining, analysing, and integrating the labelled statements, similar or related contents were merged, further refining the 216 labelled statements generated in the labelling process. In this study, 51 concepts were integrated from the 216 labelled statements. The results of conceptualization are shown in Table 4.

**Table 4:** Conceptualization of 216 tagged PE teachers’ statements.

Conceptualization	Tagged labels
PE Teacher-Led participation and role modelling in PE	PE teachers should accompany and actively partake alongside students in physical activities and athletic competitions during PE.
	A positive aspect of QPE is that teachers take the initiative to engage in PA, thus acting as role models.
	PE teachers should lead students in physical exercises in PE class.
	PE teachers should engage in PA with students in the class.
Cultivating students’ lifelong awareness of PA	QPE should cultivate students’ awareness of the need to participate actively in PA.
	Our school has hired experienced coaches from the community to provide after-school sports training services for students.
Promoting students’ holistic development	The ultimate goal of QPE is to cultivate well-rounded individuals.

For the complete data, see Supplementary Table S2.

Step 3: Categorization. Categorization was accomplished through ongoing analysis, comparison, and inductive reasoning applied to the 51 identified concepts. This process led to the extraction of categories with a strong focus on ensuring high exclusivity among them, and that each category accurately represented its constituent concepts. Finally, 12 categories were identified. The categorization results are presented in Table 5.

**Table 5:** Categorization of 51 concepts and label frequencies.

Categorizations	Conceptualization	Number of labels
Students’ development	Cultivating students’ lifelong awareness of PA	2
	Enhancing students’ critical thinking	1
	Enhancing students’ psychological benefits	4
Community-based sports engagement	Promoting community-based sports training services	2
	Organizing sports events in the community	2
	Enhancing sports equipment and facilities in the community	5
Government support for PE	Emphasizing government policy support for QPE	4
	Increasing government funding for PE	4

For the complete data, see Supplementary Table S3.

#### 2.3.2 Axial coding

Axial coding represents the second level in the grounded theory’s three-level coding framework following the initial coding stage. This phase aims to further develop and connect the concepts and categories identified during open coding. Five levels were identified in this study. The five levels were student, family, school, community, and government (see Table 6).

**Table 6:** Results of the axis coding.

Axis coding	Open coding
Student level	Students’ development
	Students’ engagement and experiences in PE
Family level	Parents’ engagement and attitude toward PE and PA
	Home-based sports resources
School level	PE teacher
	Sports facilities and equipment in the school
	PE curriculum
	School-based extracurricular PA programs
	Co-operation family-school-community in PE
	School leadership and school community support for PE
Community level	Community-based sports resources
Government level	Government support for PE

#### 2.3.3 Selective coding

Selective coding is the final stage in the grounded theory’s three-level coding process, aimed at identifying a ‘core concept’ that integrates all identified concepts (51). By conducting an in-depth analysis of the 12 categories and 5 levels established, along with the concepts and labels identified in earlier coding stages and the original interview content, the core category that emerged to encompass all other categories was defined as ‘The Practice Model of Quality Physical Education in China’.

**Figure 2:**
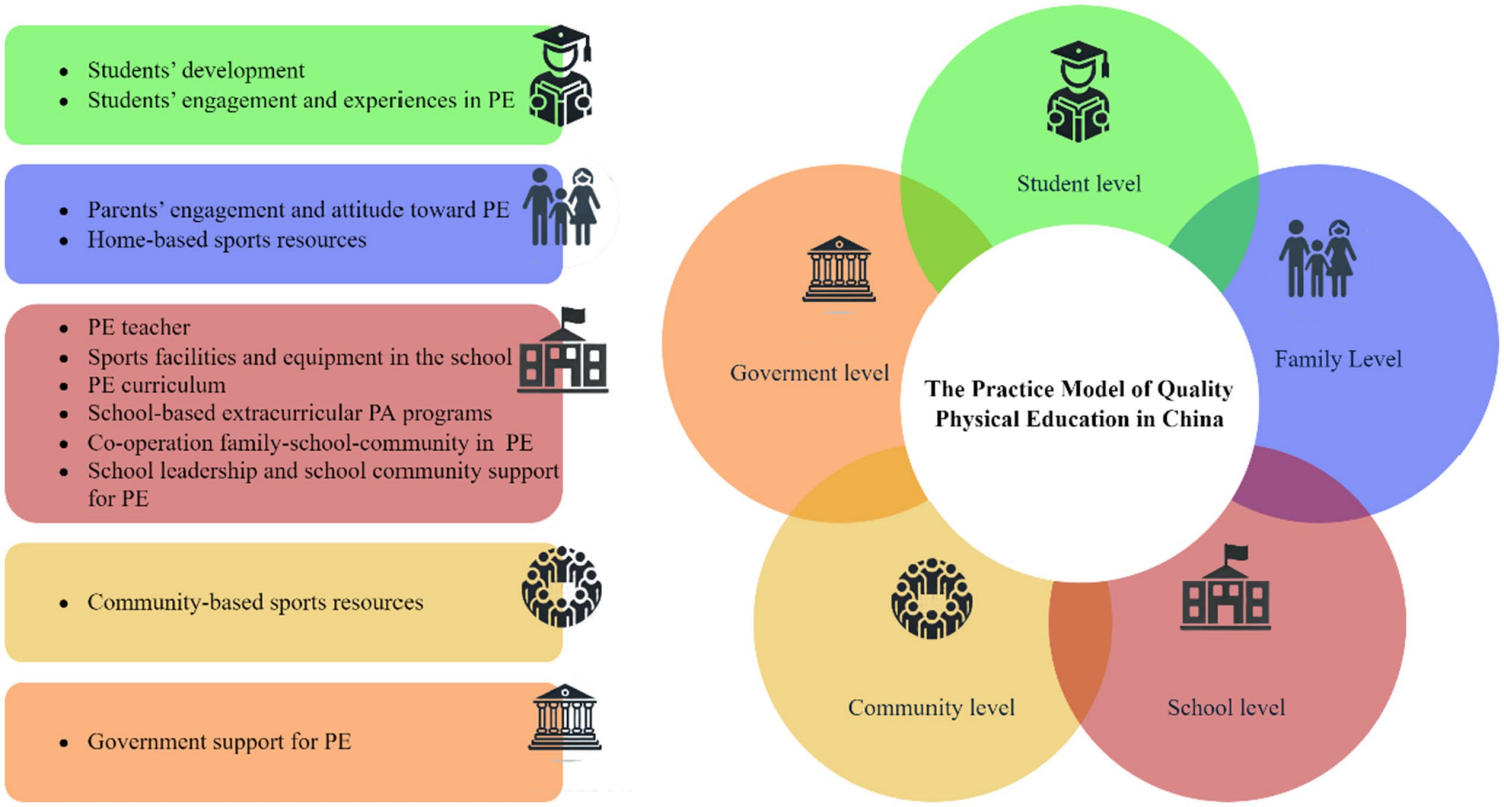
The practice model of quality physical education in China.

This study adhered to the three-level coding process in grounded theory: labelling, conceptualization, and categorization during open coding. It then advanced to levels and core concepts in axial and selective coding in second- and third-level coding. This systematic approach led to the development of a model for QPE practice in the Chinese context, comprising 5 levels and 12 categories (see Figure 2).

### 2.4 Trustworthiness

To enhance the reliability and validity of the findings, four methods were applied: (a) two coders, (b) peer feedback, (c) member verification, and (d) theoretic saturation test. First, the first and third authors independently conducted coding throughout the coding process. This approach allows for the identification of errors and omissions in the coding process while enriching the diversity of data analysis. Second, peer feedback was instrumental in mitigating biases in this study. We invited two external peer experts, specialists in the PE field, to review the final findings. This consultation was essential to ensure the validity of the results. These professionals contributed significantly by elaborating on each identified factor and conceptual domain in depth. Third, member checking was conducted by providing all participants with a summary of their analysis, along with a summary of the levels and categories contributing to QPE, based on the collective experiences of the participants. Specifically, participants were asked to evaluate how well these descriptions matched their reported experiences. We then asked them to evaluate how well these summaries reflected their own accounts. Of the 22 PE teachers, 17 agreed that the summaries were accurate. We followed up with the remaining five to clarify their concerns and address any misunderstandings. Based on their feedback, we revised the summaries to ensure each of their perspectives was accurately represented. After reviewing the updated summaries, all five teachers confirmed their agreement. Fourth, we adopted the principle of theoretical saturation. According to Wutich et al. (54), theoretical saturation occurs when no new concepts or categories emerge from additional data. In this study, we initially analysed 21 interview transcripts and reserved 1 transcript to confirm theoretical saturation. After completing the initial coding process on the 21 transcripts, we coded these final 1 interview. Since no new concepts or categories emerged, we concluded that theoretical saturation had been reached.

## 3 Results

Based on the three-level coding process of grounded theory, PE teachers’ perceptions of QPE in China can be described primarily through the overall framework of student, family, school, community, and government levels. The combination of 5 levels and 12 categories comprehensively depicts the practice model of QPE in China.

### 3.1 Student level

The constructed categorization highlights that effective QPE practice requires equipping students with comprehensive physical and health knowledge and motor skills. Moreover, ensuring students have positive and enjoyable experiences in QPE to have active lives.

#### 3.1.1 Students’ development

PE teachers emphasized that QPE can promote students’ development in physical skills, health, interests, knowledge, and social skills. The teachers identified two main categories of physical skills: motor skills, which include basic movements, such as running and jumping, that are essential for daily activities, and sport-specific skills involving complex actions requiring coordination and situational awareness. They highlighted that motor skill development is foundational for lifelong PA. Training in sport-specific skills enhances youth fitness without compromising academic performance. Additionally, PE teachers believed that QPE contributes to physical and mental health by improving strength, flexibility, and mood while reducing anxiety and depression. QPE can provide a unique environment for organized PA, alleviating academic stress and negative emotions. Fostering interest in and habits related to PA are considered essential for long-term well-being. Consequently, encouraging students to engage with PA and sports is deemed a key focus area of QPE. Finally, they agreed that QPE can promote cognitive development and social skills by offering opportunities for meaningful social interactions and reinforcing socially acceptable behaviors.

*“The main goal of the QPE program is to help students develop both motor and sports-specific skills… Through these programs, students can not only strengthen their bodies and improve their fitness but also relieve stress, achieving a balance of physical and mental health”* (Yang).

*“QPE is not just about improving students’ physical abilities; it also fosters decision-making and problem-solving skills… Students learn valuable knowledge about healthy living, which is crucial for their future… Through QPE, we aim to discover and nurture students’ interests in various sports, helping them maintain an active and healthy lifestyle beyond school”* (P. Chen).

*“For example, in a QPE basketball game, students need to constantly communicate and collaborate with their teammates to coordinate plays and achieve success. Activities like these not only enhance their physical fitness but also develop their teamwork skills”* (He).

#### 3.1.2 Students’ engagement and experiences in PE

Students’ engagement and experiences in PE are essential for the successful implementation of QPE. PE teachers observed that students with enjoyable and meaningful PE experiences are more likely to understand the importance of maintaining appropriate PA levels for their health. These students tend to develop a stronger preference for participating in PE classes and are more engaged in various PA, leading to improved physical outcomes. The quality of interactions and experiences in PE was highlighted as a key determinant of students’ perceptions of PA. PE teachers believed that positive PE experiences foster a more favorable attitude toward PA, enhance motivation, and may lead to a lifelong commitment to remain physically active.

*“High-quality PE is about making activities enjoyable for students, motivating them, and making them actively want to participate in the various activities in PE lessons”* (Yao).

*“Only when students feel that PE is a pleasure and not just a task or a requirement can they benefit from it”* (Yang).

### 3.2 Family level

This study found that the family level includes parents’ engagement and attitude towards PE and home-based sports resources. Participants also frequently mentioned the importance of family in QPE.

#### 3.2.1 Parents’ engagement and attitude toward PE and PA

PE teachers reported that parental attitudes and involvement in their children’s learning significantly influence students’ psychological well-being, attitudes, and scholastic performance, including PE. Parental engagement in children’s education facilitates positive changes and development among students. Specifically, for PE, the teachers observed that parents’ attitudes toward and involvement in PE and PA shape their children’s perceptions and interests. This influences their participation levels and potential lifelong PA habits. They believed that parental involvement in facilitating and actively participating in PA can bolster children’s enthusiasm for, and commitment to, PE and PA. This enhances the quality and efficacy of PE programs.

*“Since there is no PE test in the Chinese National College Entrance Examination, parents do not pay attention to PE and sports, which is one of the main reasons for the decline in physical fitness and PE achievement of young people as they grow older”* (Ma).

*“Some parents exercise with their children or actively participate in sports activities, which helps children develop good exercise habits and a healthy lifestyle… When parents and schools work together, students are more likely to make significant progress in PE and carry these healthy living principles into their daily lives”* (He).

#### 3.2.2 Home-based sports resources

PE teachers highlighted that home-based sports resources significantly impact students’ engagement in PA. They observed that students engage more in sports and exercise when they access several pieces of home sports and exercise equipment. PA levels were positively associated with the availability of home sports equipment (55). The teachers believed that providing home exercise resources plays a pivotal role in offering young people a consistent and familiar environment for PA. This setting facilitates the refinement of their acquired knowledge and skills. This reinforces the principles of lifelong PA instilled through PE.

*“If there is basketball or badminton equipment at home, students can practice with their family members during their free time, further improving their skills and fostering their interest in sports… Sports material support from the family can motivate students to participate more actively in physical activities, helping them develop healthy lifestyle habits”* (Feng).

### 3.3 School level

Based on this study’s findings, the school level encompasses most categories: PE teacher, sports facilities and equipment in the school, PE curriculum, school-based PA program, co-operation family-school-community in PE, and school leadership and school community support for PE. These findings align with those in previous literature, reinforcing schools’ pivotal role in the effective implementation and promotion of PE and PA programs (56, 57).

#### 3.3.1 PE teacher

The interviewed PE teachers emphasized their essential role in the success of QPE, highlighting their direct impact on students’ engagement and development. They believed that their participation and role modelling serve as powerful examples, encouraging students to value PA. According to them, QPE relies on proper teaching and guidance coupled with the continuous enhancement of their professional skills to ensure quality education. They stressed that building positive relationships with students fosters a supportive learning environment. Raising students’ awareness of responsibility is crucial for promoting healthy lifestyles. Finally, they considered high qualifications and ongoing professional development vital for delivering impactful and comprehensive QPE, supporting the holistic development of students.

*“Students learn by observing their PE teachers’ attitudes and behaviors, so teachers must model a healthy, positive lifestyle. By actively participating in physical activities and demonstrating good sportsmanship and teamwork, they motivate students to engage in exercise, appreciate its importance, and develop lasting exercise habits”* (L. Ma).

*“PE teachers should find appropriate ways to engage students in suitable physical activities so that every student can benefit from exercise… PE teachers should focus on ensuring everyone understands the skills and rules, reminding those who may be less focused to stay attentive”* (Huang).

*“Effective communication with students is paramount in PE instruction… By understanding individual abilities, respecting students’ opinions, and providing additional support when needed, teachers create a comfortable environment that fosters growth without undue pressure… Our institution values well-qualified PE teachers, requiring at least a master’s degree and a nationally recognized athlete certification for all new hires”* (Long).

#### 3.3.2 Sports facilities and equipment in the school

The quality of PE and students’ PA during their free time are mainly determined by school sports facilities and equipment. Consequently, all participants emphasized the crucial role of school facilities and equipment in implementing QPE practices. UNESCO (14) stresses the importance of safe and suitable spaces, facilities, and clothing tailored for PE, PA, and sports participants. However, a significant issue is the lack of sports facilities and equipment in Chinese primary and secondary schools (58). Interviews with PE teachers revealed that schools’ current sports facilities and equipment are inadequate for the increasing student population. Moreover, as the participants reported, the limited availability of sports equipment forces PE classes to be segmented into multiple groups to conduct exercises. This division is a direct consequence of insufficient sports facilities and equipment, which affect the efficiency and effectiveness of QPE programs.

*“The deteriorating condition of our basketball court and the overdue replacement of basic equipment like balls and racquets are major challenges in our PE teaching. Providing quality physical education is difficult when the facilities and equipment are in poor shape”* (Su).

*“Low-quality and outdated equipment not only increases the risk of student injuries but also limits the variety of sports and activities we can introduce in class. This situation restricts students’ opportunities to explore different types of physical activities and hinders the overall PE experience” (Pang).*

*“At another school, they have implemented ‘equipment boxes’ placed around the playground, stocked with various sports equipment. This system greatly improves accessibility, allowing students to easily grab what they need during PE lessons or after class, unlike centralized storage areas, which often cause delays and overcrowding”* (Tian).

#### 3.3.3 PE curriculum

A high-quality PE curriculum is essential for effectively implementing QPE practices. Integrating PE teaching content into students’ daily lives makes the learning more relevant. Adapting the curriculum to local contexts and available resources ensures that PE remains practical. This approach fosters a deeper connection between students and the subject matter. Creating an inclusive and equitable learning environment, along with diversifying and enriching the teaching content of the PE curriculum, broadens the appeal of the PE curriculum, catering to diverse student needs and interests. Aligning PE teaching content with students’ developmental stages is critical. This ensures that the PE curriculum is appropriate and beneficial for all age groups. Broadening PE assessment methods also allows for a more comprehensive evaluation of student progress and learning. Additionally, effectively structuring PE classes and creating a positive classroom atmosphere are essential for maintaining student engagement and motivation. Finally, promoting student autonomy in PE classes empowers students to take ownership of their learning, fostering a sense of responsibility and self-efficacy.

*“Students’ diverse backgrounds, abilities, and interests require a flexible PE curriculum… A rigid program can demotivate learners, whereas diverse offerings accommodate different ages, needs, and skill levels, ensuring inclusive and active participation… A rich and* var*ied PE teaching content not only improves physical fitness but also promotes teamwork, perseverance, and lifelong healthy habits.”* (Liu).

*“The current PE curriculum overemphasizes exam preparation, diminishing student engagement and motivation… A multi-week, unit-based structure covering diverse motor skills, followed by team sports or specific sports* (e.g.*, basketball, football*), *fosters skill development, cooperation, and coordination”* (Long).

*“Summative assessments* (e.g., *skills and fitness tests*) *provide standardized evaluations but overlook the learning process, individual differences, and holistic development, potentially increasing student stress and aversion to PE… In some schools, an overemphasis on motor skills from junior through senior secondary levels fails to offer age-appropriate content, leading to misalignment with students’ developmental needs.”* (XY. Chen).

#### 3.3.4 School-based extracurricular PA programs

PE teachers highlighted that integrating various PA is essential for a comprehensive QPE experience. In China, daily PA routines have been institutionalized and integrated into students’ daily lives. Specifically, a structured 30-min PA program is mandated daily for primary and secondary schools, according to the government’s directive (35). However, interviews indicate that the routine of running 10 laps every morning is monotonous, highlighting a lack of diversity in PA routines, as noted by Kong (59). PE teachers suggested that extracurricular sports and activities, including after-school training, sports teams, competitions, and events, can enhance the PE experience by providing diverse opportunities beyond the standard curriculum. They believed that these school-based extracurricular activities help students develop various physical skills, foster a lifelong interest in staying active, and improve their fitness, concentration, and academic performance. PE teachers observed that students are more engaged in PA when offered a range of extracurricular options. Therefore, they emphasized promoting diverse extracurricular activities and ensuring regular opportunities for PA throughout the school day, both before and after school, as critical to achieving a holistic QPE program.

*“We allocate 30 min daily for students to engage in physical activities like running and aerobics. This daily PA routine is considered a crucial component of high-quality PE because it cultivates students’ physical skills and habits and educates them on the value of regular daily exercise”* (Tan).

*“Our school has introduced after-school sports training services. This post-class sports service is vital for high-quality PE, offering a platform for students to engage in sports activities beyond academic learning. This policy emphasizes PE’s pivotal role in students’ holistic development, enhancing their physical health and quality of life”* (Li).

*“By incorporating various sports activities, we honor and leverage our students’ diverse interests and talents, significantly enhancing their health, self-esteem, and social competencies. This diversity is integral to a QPE because it addresses and celebrates individual student profiles, promoting a well-rounded, confidence-building educational experience”* (Sheng).

#### 3.3.5 Co-operation family-school-community in PE

PE teachers in this study highlighted that the key to achieving QPE lies in establishing an effective collaborative framework centered around schools. This framework should enable schools, families, and communities to work together based on student-specific needs. Epstein (60) posited that students’ learning and development occur within intersecting families, schools, and community environments. She emphasizes the ‘overlapping’ effect of these three spheres in education, highlighting the comprehensive benefits and complementary roles of family, school, and community in their functions and contributions. However, in China, PE is primarily conducted by schools, with limited collaboration with families and communities (59). This lack of co-operation restricts family and community involvement in students’ PE, resulting in inadequate PA opportunities outside school hours and a failure to meet individual students’ diverse needs.

*“Active partnerships with students’ families and the wider community are key factors in improving the quality of PE. They extend the reach of PE beyond the school setting, promote a more holistic approach to student health and wellness, and broaden our message about the importance of PE as a social endeavor rather than a separate school activity”* (Yang).

#### 3.3.6 School leadership and school community support for PE

The interviewed PE teachers emphasized the crucial role of school principals and leaders in achieving high-quality PE. They highlighted that prioritizing PE within the curriculum, allocating sufficient funding to the PE department, and engaging in collaborative discussions on teaching assignments are essential factors. The teachers noted that the qualifications and experiences of school principals significantly affect their support for QPE. Principals who value personal development tend to prioritize teacher welfare, which enhances PE teaching and learning. The teachers also observed that physically active principals demonstrate greater commitment to quality PE programs than less active ones. Additionally, they mentioned that the past experiences of non-PE teachers and peers in PA, sports, and PE can influence their attitudes toward PE, subsequently affecting their students’ participation in PA and PE. Beyond these specific factors, the teachers stressed that an environment filled with encouragement and a positive climate can bolster individuals’ self-perceptions and self-efficacy, guiding their choices and behaviors.

*“Our principal has emphasized the importance of PE in the school’s development plan. He has increased investment in sports facilities and established a dedicated PA Week to encourage student participation”* (Meng).

*“The classroom teacher’s attitude toward PE and sports activities is crucial for quality PE. Their support and active participation not only spark students’ interest in sports but also create a positive and healthy learning environment. Therefore, I believe the classroom teacher’s attitude is a key factor in achieving QPE”* (Dai).

*“There is a student who excels in basketball. He not only actively participates in basketball competitions both within and outside the school but also takes the initiative to organize and lead training sessions with his classmates. His enthusiasm and positive attitude have inspired many of his peers, creating a vibrant sports atmosphere in the class”* (Liang).

### 3.4 Community level

The qualitative analysis in this study demonstrates that the community level comprises community-based sports resources.

#### 3.4.1 Community-based sports resources

Community-based sports resources serve as a tangible manifestation of societal priorities, values, and a commitment to enhance the physical well-being of the youth and nurture their development. Abundant community sports resources often include readily available and accessible facilities and equipment. They provide PA programs and sports training organizations that cater to diverse age groups and interests. These resources also play a pivotal role in promoting a culture of active living (61–63). QPE can be effectively augmented in school settings by promoting extracurricular PA and disseminating information on community-based sports initiatives (64). Opportunities also exist for community collaboration to furnish quality PA resources by constructing new facilities and enhancing current ones (65). Additionally, the interviews with PE teachers in this study indicated that the community is the primary living space for students. Sports resources ensure that students exercise in a safe environment after school, which is essential for fostering their sports skills and an overall active lifestyle.

*“The sports training organizations in the community offer training methods and standards that differ from the school PE curriculum, providing students with more professional and systematic training. The training students receive in these organizations enhances their physical capabilities and helps them achieve higher levels of motor technique and sport strategy. This complementary approach ensures that students develop holistically, not just limited to what the school PE curriculum offers”* (L. Ma).

### 3.5 Government level

Governmental entities must proactively empower schools to establish conditions that foster health promotion and support PA (66). The government’s key role is to improve PE quality (14). Based on this study’s findings, the government level includes government support for PE.

#### 3.5.1 Government support for PE

PE teachers argued that the development of QPE is a complex and dynamic process requiring long-term planning and government intervention. They viewed government support as the central driving force in enhancing PE across schools. The teachers highlighted that governmental entities are pivotal in helping schools create health-enhancing environments that boost students’ PA levels. Specifically, they noted that government support provides ample PE facilities, such as open fields, and playgrounds. The teachers suggested that the government can create a supportive environment for QPE by providing policies, funding, and regulations. They identified several indicators of effective government intervention, including PE quality, adequate equipment, facility standards, support from educational departments, manageable class size, and opportunities for professional development. According to the teachers, these factors are essential for delivering impactful and comprehensive QPE, ultimately supporting students’ holistic development.

*“When the government introduced the ‘Health and Fitness for All’ and ‘Health First’ policies, it was a game-changer for us. These policies emphasized the importance of physical health among the younger generation and allowed us to support students who were talented in unconventional sports, fostering a sense of inclusion and diversity in our PE approach”* (Liu).

*“When our school became a beneficiary of the ‘Priority School in Municipality’ grant, we received a financial boost that enabled us to revolutionize our sports facilities. We acquired equipment ranging from basic items like balls and nets to more sophisticated gear such as heart rate monitors and resistance training tools.”* (Y. Ma).

## 4 Discussion

This study’s objective was to explore PE teachers’ perceptions of QPE in China using grounded theory. We aimed to develop a model of QPE in China to improve adolescents’ physical health. The findings revealed that QPE in China can be categorized into 5 levels, 12 categories, and 51 concepts.

PE teachers expect QPE to foster students’ holistic development and promote their physical, emotional, social, and cognitive growth. In China, concerns over declining PA levels among children and adolescents have intensified, with more than 87% of young people not meeting the World Health Organization guidelines for recommended PA levels (67, 68). This downward trend in PA has contributed to the deterioration of students’ health (33). In response to this challenge, the curriculum reform of 2022 underscored the role of PE in cultivating students’ awareness of health and safety, fostering sustainable healthy lifestyle habits, enhancing both physical and mental well-being, and supporting their holistic development (37). PE teachers involved in this study further highlighted the significance of these reforms in promoting students’ holistic development, which is a key aspect of QPE. Holistic development encompasses physical, emotional, social, and cognitive growth. Moreover, these findings align with those of Western national organizations (21, 23, 24), which also suggest that QPE promotes students’ holistic and sustainable development by fostering physical, emotional, and social growth, equipping them with the skills and confidence for PA.

This study found that delivering QPE requires a holistic approach that involves multiple stakeholders such as students, families, schools, communities, and governments. This multi-layered involvement is supported by previous research. Cale and Harris (69) argued that the PE curriculum alone cannot address the broader environmental factors influencing PA behaviors. Empirical research demonstrated that traditional PE often fails to significantly contribute to overall PA levels. For instance, Iglesias et al. (70) in a review of 224 studies involving over 80,000 students from more than 450 primary and secondary schools worldwide, found that students consistently fail to meet the recommended guideline (71) of engaging in MVPA for at least 50% of PE class time regardless of country, educational stage, gender, or methods used to measure MVPA. In response, O’Connor et al. (72) applied a social-ecological framework to PE, recognizing that an individual’s movement experiences are shaped by multiple layers of influence, such as intrapersonal factors (biology and psychology), interpersonal relationships (social norms and culture), environmental settings, and policies. Moreover, Epstein’s (60) overlapping influence domain theory posits that students’ learning and development occur within the intersecting environments of family, school, and community. This holistic framework allows PE to account for the wider social contexts that either support or hinder lifelong engagement in sustainable PA. Similarly, Ho et al. (52) identified eight dimensions of QPE: skill development and bodily awareness; facilities and norms in PE; quality teaching of PE; plans for the feasibility and accessibility of PE; social norms and cultural practice; governmental input for PE; cognitive skills development; and habituated behavior in physical activities, all of which are essential for fostering QPE practices. Kingston et al. (73) emphasized the importance of government leadership, school management, teacher involvement, parental support, and community partnerships to ensure the successful implementation of QPE. These studies align with the present study, underscoring the need for a multi-layered approach to QPE involving stakeholders at all levels, ranging from students to governments. Notably, New Zealand and Australia have implemented such collaborative models to enhance QPE by integrating stakeholders across different levels, such as students, schools, and the government (74, 75), thereby promoting adolescents’ health.

This study’s findings of 5 levels and 12 categories are interconnected and mutually influential. For example, students’ development is shaped not only by their engagement and experience in PE, but also by external factors such as family support (73), school resources (76), and community engagement (14). Parents’ involvement and positive attitudes toward PE are essential, as they encourage students to participate more actively in PE classes (77). Additionally, the availability of sports resources at home, such as equipment or space, provides opportunities for sustainable exercise outside school (55). Schools play a central role in fostering student development, engagement, and experience by providing a structured PE curriculum, qualified PE teachers, and proper sports facilities and equipment (78). The wider community contributes by offering resources and creating a culture that encourages sustainable PA (79). Government support ensures that schools and communities have the necessary infrastructure, training, and resources to implement effective QPE programs (15).

Three distinct characteristics of QPE practice in China emerged from this study. First, PE teachers address the important role of the government due to China’s governmental framework and centralized approach to education policy. The government is central to shaping and implementing national educational policies, including those for PE and adolescents’ health development (33). Stakeholders align their efforts with these policies, positioning the government as the primary driver of QPE. Moreover, the government provides funding, infrastructure, sports facilities and equipment, and teacher training. This support ensures the consistent and effective delivery of QPE across the country. Second, the school level emerged as the important participant in delivering QPE. It encompasses six categories, which are the most among all the levels. This indicates that schools play a central role in implementing QPE practices. Studies have shown that school resources, management, and the quality of PE teachers significantly impact the effectiveness of PE programs (14). This emphasis on schools underscores their pivotal role in shaping students’ PE experiences and PA participations. Third, unlike in some Western contexts, the family level plays a more pronounced role in QPE practices in China. Cultural differences between East and West may account for this variation (80). In Chinese culture, family involvement in education is highly valued, and parental support is considered essential for student success (81). This contrasts with Western models (21, 23, 24), in which family involvement in PE may be less emphasized. Recognizing this cultural nuance adds depth to our understanding of how QPE can be tailored to various societal contexts.

### 4.1 Implications

The findings of this study have significant implications for PE in China and other countries. First, by uncovering the five levels and 12 categories of QPE practices specific to the Chinese context, this study implies that generic, one-size-fits-all QPE models may not be effective in all settings. This suggests that educational policymakers should contextualize QPE frameworks, rather than adopting Western models without adaptation.

Second, the practical framework we propose offers valuable guidance for Chinese PE teachers and stakeholders. By utilizing the identified five levels and 12 categories, educators can improve PE quality, foster cross-sector collaboration, and support sustainable development within the educational system. This approach provides a concrete pathway for practitioners to implement changes that align with the unique characteristics of China’s education system, thereby promoting increased PA participation among adolescents and enhancing their physical health.

Third, by adopting this context-specific model, stakeholders can look beyond the traditional components of sport, health, and exercise. They can act as catalysts for change within the PE curriculum, schools, families, communities, and governments, extending their impact beyond scheduled PE sessions. This study advocates for multi-layered support for PE to transform its role from merely providing activities to acting as a connector among families, schools, communities, and governments. This transformation contributes to the creation of a sustainable educational system.

Finally, the QPE model allows for monitoring progress and aligning the curriculum with national goals. Although it was developed in China, the five levels and 12 categories draw on broader pedagogical and administrative principles, making them adaptable to diverse educational systems. Countries can tailor these categories to local policy frameworks, teacher development standards, and resource constraints. This flexibility ensures that the QPE approach remains responsive to different cultural and institutional contexts, enabling stakeholders to implement QPE initiatives that align with their specific needs. As a result, the model has the potential for application beyond China and could inform PE programs in other nations seeking to enhance the quality and consistency of physical education instruction.

### 4.2 Limitations and future study

Our study has several limitations. First, due to the constraints imposed by the COVID-19 pandemic, our interviews were conducted online, which is a limitation in capturing nonverbal communication cues (82). Although this study employed qualitative methods to examine the QPE practice model in China, it did not include interviews with policy makers, teachers from primary and university, or in other countries. This may limit the generalizability of our findings across different educational contexts and geographic areas. Furthermore, the specific procedures for implementing QPE remain unclear.

Owing to these limitations, further improvements can be made in future studies. Future research could further explore PE teachers’ experiences and perceptions in different educational stages, such as primary school and university PE teachers and other PE professionals, and consider expanding the sample size to encompass various regions. These distinct groups and regions offer unique perspectives on QPE, as their interactions and experiences in PE may vary. This could aid in developing a more comprehensive understanding of various factors and their effects on QPE. Building on the levels and categories identified in this study, future research could adopt quantitative methodologies to investigate the implementation of QPE practices. For instance, a questionnaire based on this study could be developed to assess the practical implementation of QPE.

## 5 Conclusion

This study developed the QPE practice model using three-level coding grounded in the principles of grounded theory. Through participant interviews, 51 concepts and 216 labels were identified, resulting in five levels and 12 categories. It addresses a critical gap in Chinese QPE research by capturing PE teachers’ perceptions and proposing a holistic approach that positions PE as a connector among families, schools, communities, and governments to promote adolescent health. However, the study did not include policy makers, teachers from primary schools or universities, or participants from other countries. Future studies could expand on this research by exploring PE teachers’ experiences across various educational stages in different countries. In addition, employing quantitative methodologies, such as a developing a questionnaire, could provide valuable insights into the implementation of QPE practices.

## Data Availability

The original contributions presented in the study are included in the article/Supplementary material, further inquiries can be directed to the corresponding author.
